# Development of an aptamer-functionalized sorbent for the selective and specific extraction of lead from environmental and biological samples

**DOI:** 10.1007/s00216-026-06561-8

**Published:** 2026-05-20

**Authors:** Alice Taxil--Paloc, Fanny Gignac, Nathalie Delaunay, Valérie Pichon

**Affiliations:** 1 Department of Analytical, Bioanalytical Sciences, and Miniaturization, UMR 8231 Chemistry, Biology and Innovation, ESPCI Paris, PSL University, CNRS, 10 Rue Vauquelin, 75005 Paris, France; 2 Sorbonne Université, 75005 Paris, France

**Keywords:** Solid-phase extraction, Aptamers, Oligoextraction, Lead, ICP-MS

## Abstract

An oligonucleotide-based sorbent was developed to enable the selective extraction of lead ions from serum and water samples. Two oligonucleotide sequences, previously reported in the literature for their high affinity toward Pb(II), were grafted onto CNBr-activated Sepharose, producing sorbents suitable for solid-phase extraction. Grafting efficiencies ranged from approximately 50 to 70%. Subsequent optimization focused on adjusting the percolation and washing conditions to ensure preferential retention of Pb(II) on the specific aptamer-modified supports, followed by controlled elution. The elution kinetic was studied to minimize the elution volume. Under optimized conditions, high selectivity was obtained, with mean extraction recoveries of 83 ± 2.7% and 79 ± 8% (*n* = 3) for the specific sorbents, compared to only 7–10% for the control sorbents. Other cations showed recoveries below 15%, confirming the strong specificity of the developed sorbents. The capacity of the most efficient sorbent was estimated at approximately 600 ng per 30 mg of support (≈30 µg/g), which is suitable for the selective extraction of trace amounts of Pb(II) from serum or water samples. Applying this support to real samples made it possible to determine Pb(II) levels of 6.3 µg/L for tap water, 2.7 µg/L for the Seine River water, and 87 µg/L for digested serum samples, while reducing matrix effects and minimizing ICP-MS instrument clogging.

**Graphical abstract:**

**Figure:**
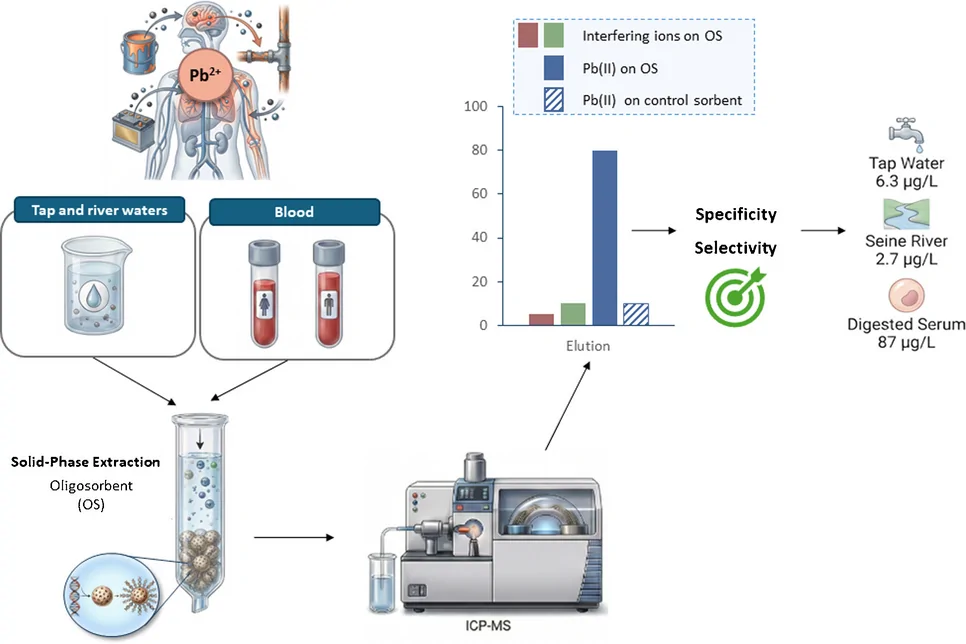

**Supplementary Information:**

The online version contains supplementary material available at https://doi.org/10.1007/s00216-026-06561-8.

## Introduction

Contamination by heavy metals, especially lead, is increasingly concerning due to its health impacts. There are many sources of lead exposure, from paints and batteries to water pipes. Lead poisoning is known to cause irreversible physical and intellectual retardation in children, including language disorders, slowed growth, motor difficulties, and hearing problems [1]. To reduce the risk of lead ingestion, regulatory limits have been established for drinking water, setting the maximum allowable concentration at 10 µg/L [2]. Environmental quality standards have also been defined, specifying an annual average limit of 1.2 µg/L and a maximum admissible concentration of 14 µg/L [3]. To monitor the exposure of populations and industrial workers, some countries have set lead concentrations to be found in blood, which is 300 µg/L for women and 400 µg/L for men in France [4]. It is therefore necessary to have methods available for determining the amount of lead present in these different types of complex samples.

For such complex samples, a pretreatment step is necessary to limit matrix effects and interferences during quantification. Solid-phase extraction (SPE) is the commonly used method [5]. Chelating resins are widely employed for Pb(II) extraction [6] but lack specificity, causing co-extraction of other cations. To overcome this limitation, expensive instruments like ICP-MS are often employed to quantify lead. Alternatively, improving extraction specificity can reduce the need for such costly instrumentation. Consequently, new specific and selective sorbents have been developed, such as ion-imprinted polymers (IIPs) [7]. IIPs are synthetic polymers containing cavities that selectively trap target ions. Although their potential is well established, their synthesis remains complex. For this reason, some researchers have turned to aptamer-based sorbents.

Aptamers are single-stranded DNA or RNA (typically 20–110 bases) with high specificity and affinity for selected molecules or ions. They are identified by a complex process called SELEX (Systematic Evolution of Ligands by EXponential enrichment) [8]. Aptamers immobilized on solid supports have been widely reported for the solid-phase extraction, i.e., oligoextraction, of molecules [9], from small drugs to proteins, and more recently, aptamer-based sorbents have also emerged as promising tools for selective extraction applications, including metal ions, although their use for ion extraction remains limited. Regarding Pb(II), specific sequences have been identified and used for the development of sensors [10–24]. Concerning extraction, some developments have been carried out implementing oligoextraction only in the dispersive mode [25–27]. Although these works demonstrated the capability of certain sequences to retain Pb(II), a certain amount of data has not been investigated. One of the missing studies in these works is the study of the selectivity of the extraction process, meaning the comparison of extraction performances between a specific support and a control support. It has been recently shown in a previous study by our team, dealing with the development of an oligosorbent for cadmium, that this parameter is crucial for selecting a support whose retention is ensured by the sequences and not by non-specific interactions [28]. Furthermore, a study of competition between lead and other ions was done by the authors to determine whether the presence of other ions had an impact on lead determination, but the study of the retention of these ions on the support, i.e., of the specificity of the support, was not carried out. However, specificity and selectivity are particularly important parameters if we wish to use these supports in the future in association with detectors that are less specific but less expensive than ICP-MS. Regarding the sequences described in literature, most of them contain the guanine repeat motif that enables folding into a G-quadruplex structure. Indeed, this structure is well-known for strongly interacting with lead ions, with the latter positioning itself at the center of the structure to stabilize it [29]. However, several sequences enabling this folding have been identified, without the performance of each of them having been compared.

The objective of this work was to investigate the potential of aptamers to extract lead ions from complex samples, while ensuring the contribution of aptamers to the extraction process, in terms of specificity and selectivity, by studying in parallel the behavior of lead and of other cations on control sorbents. Two sequences among those reported in literature [10, 27] were therefore chosen to be grafted onto CNBr-activated Sepharose and packed in a cartridge. In addition to these oligosorbents (OSs), control sorbents were also prepared to study the selectivity of the supports, i.e., the ability of the specific sequence to retain the target and not the controls. After measuring the grafting yields of oligonucleotides onto CNBr-activated Sepharose, the performances of the two lead-specific OSs were evaluated and compared in terms of selectivity and specificity toward other cations. Once the extraction protocol was optimized, the capacity of the supports was determined. Finally, the optimized extraction protocol was applied to the extraction of Pb(II) tap water, river water, and serum samples prior to analysis by ICP-MS.

## Experimental

### Reagents and samples

Metal standards (in 2% nitric acid), Trizma hydrochloride (Tris–HCl; ≥ 99%), ethylenediaminetetraacetic acid tetrasodium salt (EDTA; ≥ 99%), sodium phosphate dibasic (≥ 99%), sodium dihydrogen phosphate dihydrate (≥ 98%), sodium acetate (≥ 99%), sodium chloride (≥ 99%), magnesium chloride (≥ 98%), Trizma buffer (≥ 99.9%), 65% nitric acid (Suprapur®, triethylamine (TEA; ≥ 99.5%), glacial acetic acid (100%), 2-(N-Morpholino)ethanesulfonic acid (MES; ≥ 99.5%), 4-(2-hydroxyehtyl)piperaine-1-ethanesulfonic acid (HEPES; ≥ 99.5%), potassium chloride (≥ 99.5%), sodium perchlorate (≥ 98%), sodium nitrate, human serum, and CNBr-activated Sepharose (4B, 90 µm) were obtained from Sigma-Aldrich (Saint-Quentin-Fallavier, France). Potassium phosphate monobasic was obtained from VWR (Rosny-sous-Bois, France). Acetonitrile (ACN) was obtained from Carlo Erba (Val-de-Reuil, France). Hydrochloric acid 32% was obtained from Merck (Darmstadt, Germany). High-purity water was obtained using a Milli-Q purification system (Millipore, Saint-Quentin-en-Yvelines, France). The ICP-MS was daily tuned using an Agilent tuning solution (Ce, Co, Li, Mg, Tl, and Y at 1 µg/L in 2% HNO_3_ (Les Ulis, France)). 5′-amino-modified with C_6_ spacer arm DNA oligonucleotides (sequences 5′-GGGTGGGTGGGTGGGT-3′ (named AP1), 5′-GGTTGGTGTGGTTGG-3′ (named AP2), 5′-TTTTTTTTTTTTTTT-3′ for control 1 (named C1), and 5′-GTGTGTGTGTGTGTG-3′ for control 2 (named C2)) were synthetized and purified by Reverse-Phase High-Performance Liquid Chromatography (RP-HPLC) by Eurogentec (Angers, France). A pooled human serum was purchased from Pan Biotech (Aidenbach, Germany).

### ICP-MS

The ICP-MS analytical method was based on previously established procedures developed by our group [28]. The quantification of ion concentrations in the different solutions was achieved using an Agilent 7850 ICP-MS system (Agilent Technologies, Les Ulis, France) equipped with a glass MicroMist nebulizer, a quartz Scott-type spray chamber, and an ASX-500 autosampler. The instrumental settings were as follows: RF power of 1550 W, sampling depth of 8 mm, plasma gas flow rate of 15 L/min, auxiliary gas flow rate of 0.9 L/min, and helium flow rate of 4.3 mL/min. To ensure stable analytical conditions, a daily performance check was performed by monitoring ^7^Li, ^89^Y, ^205^Tl, the CeO/Ce ratio, and the Ce^2^⁺/Ce ratio. These ratios were maintained below the acceptance limits of 1.2% and 2%, respectively. Each measurement consisted of five replicates, with 100 sweeps and a 1-s integration time per replicate. The isotopes analyzed were ^59^Co, ^60^Ni, ^63^Cu, ^66^Zn, ^111^Cd, ^206^Pb, ^207^Pb, and ^208^Pb. Prior to analysis, all samples were diluted tenfold in 2% HNO₃. Calibration curves were generated daily to calculate ion concentrations, covering the range from 50 ng/L to 10000 µg/L. The limit of quantification (LOQ) was assessed by analyzing ten blank 2% HNO₃ solutions, applying the equation given in Supplementary Equation S1. The resulting LOQ for Pb(II) was 45 ng/L. LOQ values for the other interfering ions were determined in the same manner and are presented in Table S1 (Supplementary Data).

### Oligosorbent synthesis

The procedure for immobilizing aptamers on CNBr-activated Sepharose was previously described by our group for cadmium ions [28]. Briefly, aptamers were dissolved at 1 g/L in 200 mM Na_2_HPO_4_ + 5 mM MgCl_2_ (pH 8), denatured at 75 °C for 5 min, and allowed to cool for 30 min. CNBr-activated Sepharose (30 mg) was swollen with HCl and rinsed with ultra-pure water, and then mixed with 150 µL of aptamer solution overnight at room temperature. The resulting OS was packed into a 1-mL SPE cartridge and washed three times with 1 mL 200 mM Na_2_HPO_4_ (pH 8), and the remaining active groups were blocked by percolating 0.1 M Trizma for 2 h. The OS was then washed three times alternately with 2 mL acetate buffer (0.1 M acetate + 0.5 M NaCl, pH 4) and 2 mL Trizma buffer (0.1 M Trizma + 0.5 M NaCl, pH 8), and conditioned with 5 mL ultra-pure water. The oligosorbents synthesized are named OS1 (AP1 sequence), OS2 (AP2), and the non-specific controls OSc1 (C1) and OSc2 (C2).

### RP-HPLC–UV analysis of aptamer

Determination of the grafting yield was performed by quantifying the amount of aptamer remaining in the solutions collected after the grafting step, using a procedure derived from a previous work of our group [28]. The analysis was carried out by ion-pair reverse-phase HPLC with UV detection (Agilent 1200 Series HPLC system, equipped with a 1100 Series autosampler and a 1200 Series UV detector). The detection wavelength was set at 260 nm. Chromatographic separation was achieved on a Kinetex core shell C18 column (150 × 3 mm i.d., 5 µm, 100 Å; Phenomenex, Le Pecq, France) maintained at 50 °C, and protected by a precolumn filter (0.5 µm frit, 2.39 × 1.65 mm; Upchurch Scientific, Oak Harbor, WA, USA). The mobile phase consisted of water containing 0.1 M triethylammonium acetate (TEAA, prepared from triethylamine and acetic acid, pH 7) and acetonitrile. The gradient applied adapted from previous work [28] started with 7% ACN for 5 min, increased linearly to 15% over 15 min, held at 15% ACN for 5 min, and finally returned to 7% ACN for re-equilibration over 10 min. The flow rate was fixed at 200 µL/min, and the injection volume was 20 µL. Calibration curves for each aptamer sequence were established between 0.1 and 10 µg/mL in 200 mM Na₂HPO₄ and 5 mM MgCl₂ buffer, adjusted to pH 8.

### Extraction procedure

#### Pure media

To determine the appropriate percolation medium, several tests with different solutions spiked with Pb(II) and other cations were performed. Slight modifications were made to the procedures (in terms of volume and pH of the percolation and washing solution) to ensure optimal retention and recovery of Pb(II). Details of the procedures used to generate each SPE profile are provided in the figure captions, with all associated figures available in the Supplementary Data (S2-S11).

The final extraction procedure for extracting Pb(II) from spiked pure medium on the OS was as follows: conditioning with 5 mL of MgCl_2_ (5 mM, pH 6), percolation of 1 mL of MgCl_2_ (5 mM pH 6) spiked at 30 µg/L with Pb(II) and eventually other ions (each at 30 µg/L) through the OS, washing with 5 × 1 mL of MgCl_2_ (5 mM, pH 6), elution with 3 × 250 µL of 1 mM EDTA. All these steps were performed with solutions maintained at 4 °C except for elution which was conducted at room temperature. Each SPE fraction (percolation, washing, and elution) was then diluted 1/10 in 2% HNO_3_ for ICP-MS analysis. The capacity of OS was determined by percolating 1 mL of MgCl_2_ (5 mM, pH 6) containing increasing amounts of Pb(II) from 30 to 900 ng, and the recovered elution fractions were further diluted in 2% HNO_3_ while applying a dilution factor appropriate to the amount of Pb(II) introduced to fall within the calibration range of the ICP-MS analysis.

#### Real water samples

The tap and Seine water samples from Paris were collected in polypropylene (PP) bottles and stored at 4 °C until use. For Seine water, samples were first filtered using a 0.2-µm membrane (cellulose acetate, Sartorius Stedim biotech, Aubagne, France). Subsequently, 0.5 mL of a 500 mM MgCl_2_ solution having a pH of 6 adjusted with 2% HNO_3_ was added to 49.5 mL of sample after spiking with Pb or not to obtain a final concentration of 5 mM MgCl₂. The optimized oligoextraction protocol was applied to 1 mL of the water sample. After oligoextraction, the elution fractions were diluted 1/10 in 2% HNO_3_ and analyzed by ICP-MS. The determination of lead concentrations was done thanks to a calibration curve daily performed (an example is provided in Fig. S1). To study the potential of oligoextraction, 1 mL of water was directly diluted (1/10 in 2% HNO_3_) and analyzed by ICP-MS (without OS).

#### Serum samples

This sample treatment workflow was developed based on earlier work performed in our team [28]. Briefly, serum samples were first subjected to an acidification step to remove proteins that may complex with lead and clog the SPE cartridges. For this purpose, 500 µL of serum, either spiked or untreated, were placed in an Eppendorf tube and combined with 30 µL of 65% HNO_3_. After centrifugation for 10 min at 12,000 rpm, the supernatant was collected and centrifuged a second time under identical conditions. The clarified supernatant was then transferred to a 10-mL polypropylene tube and brought to a final volume of 10 mL with 9.5 mL of MgCl_2_ solution (5 mM MgCl_2_, pH 6). The pH was adjusted to 6 using 2 M NaOH. One milliliter of this pretreated serum was then processed using the optimized oligoextraction protocol. Following oligoextraction, the elution fractions were diluted 1/3 in 2% HNO_3_ prior to ICP-MS analysis. To assess the performance of the method, serum samples were spiked at concentrations of 100 and 300 µg/L of Pb(II), and the concentrations measured after oligoextraction were compared with those obtained from samples that underwent only acid digestion followed by dilution (overall dilution factor of 60, chosen to match the final dilution after extraction). Three replicates were performed for each spiking level.

## Results and discussion

### Oligosorbent synthesis and grafting yields

Sequences reported in literature as specific for Pb(II) were selected: AP1 (5′-NH_2_-(CH_2_)_6_-GGGTGGGTGGGTGGGT-3′) consists of four repeats of a 3-guanine motif separated by thymine [10–17, 25], while AP2 (5′-NH_2_-(CH_2_)_6_-GGTTGGTGTGGTTGG-3′) contains repeats of 2 guanines separated by one or more thymines [20, 22–24], forming the G-quadruplex motif. Two control sequences were designed to assess selectivity. Due to the repeating patterns in AP1 and AP2, scrambled sequences [30, 30] could not be used, as randomization would create unintended G-G motifs. Instead, a poly-T sequence preventing G-quadruplex formation was selected as control 1 (C1) and alternates guanine and thymine, preventing G-quadruplex formation while maintaining similar nucleotide composition, as control 2 (C2). The four sequences were grafted onto CNBr-activated Sepharose to obtain OS1 (grafted AP1 sequence), OS2 (grafted AP2 sequence), and the non-specific controls OSc1 (grafted C1 sequence) and OSc2 (grafted C2 sequence). Grafting yields were determined by quantifying non-grafted aptamers in the grafting and washing solutions via ion-pair RP-HPLC–UV. They range from 53 to 71% (see Table 1), which are values slightly higher than the ones obtained previously by grafting other aptamers onto the same CNBr-Sepharose (20–63%) [28, 30], and may be related to the small size of the aptamer sequences, which facilitates their grafting. In addition, the sequences were grafted multiple times by different experimenters at several-month intervals, and the resulting grafting yields were highly consistent across repetitions, with grafting yields of 60 ± 11% (*n* = 3) for OS1, as shown in Table 1. The theoretical capacity of each OS, i.e., the amount of Pb(II) that could be retained selectively, was then calculated assuming a 1:1 aptamer-to-lead stoichiometry. The calculated values are reported in Table 1 and are sufficient for extracting traces of Pb(II) from real samples.

**Table 1:** Grafting yields measured for each synthesized OS and their corresponding theoretical capacity values estimated for SPE cartridge containing 30 mg of grafted Sepharose

Oligosorbent	Grafting yields (%)	Theoretical capacity (ng)
OS1	60 ± 11 (*n* = 3)	3546 ± 605 (*n* = 3)
OS2	54 ± 1 (*n* = 2)	3422 ± 82 (*n* = 2)
OSc1	54 ± 11 (*n* = 3)	-
OSc2	53	-

### Selection of the extraction media

The first step of an SPE procedure is sample percolation, during which the analyte should be retained. Selecting a medium that promotes Pb(II) retention on OS1 and OS2 is therefore essential. Using the selection buffer as the percolation medium appears interesting because it should favor the binding of the target by the aptamer during the selection procedure. However, for the sequence selected for this study, the SELEX conditions were not reported in the literature, preventing its use. Since the aptamers used here were originally described for the determination of Pb(II) by aptasensors, it was assumed that the same type of media involved in these studies could also promote Pb(II) retention during the SPE procedure. However, the aqueous media reported in these studies vary widely and were not always clearly described. For the two aptamer sequences used in this work, several aqueous media were reported for Pb(II) dispersive extraction [25–27] and aptasensors [10–24]. Consequently, Pb(II) retention was evaluated in these different buffers (PBS, HEPES, MES) and salt solutions (NaCl, MgCl₂, NaNO₃) to assess both specificity and selectivity.

Selectivity was evaluated by comparing Pb(II) retention on the specific aptamer support with that on the control support C1. Specificity was evaluated by studying the retention of Cd(II) and Co(II) introduced at the same concentration as Pb(II). They were selected as interfering ions because of their chemical similarities to lead and their frequent co-occurrence in environmental matrices. Both elements form divalent cations with comparable behaviors, which can challenge the specificity of sorbents. Including Co and Cd ions in this study of specificity therefore provides a stringent assessment of the material’s ability to discriminate Pb from other competing metal ions commonly encountered in real samples.

After percolation of the spiked aqueous solutions made of buffers or salts, the OSs were washed with the same aqueous medium to remove weakly bound ions, and then retained ions were eluted with 1 mM EDTA, selected for ion elution because of its strong chelating ability without degrading the sorbent. Ion quantification was performed by ICP-MS in each percolation, washing, and elution fraction after dilution with 2% HNO₃. Table 2 summarizes the results (see Figures S2–S10 for detailed SPE profiles in supplementary data).

**Table 2:** Evaluation of Pb(II) retention during percolation, specificity (Pb(II) vs Cd(II) and Co(II)), and selectivity (Pb(II) on the specific OS vs control OSc1/2) for all tested percolation media. Detailed results are shown in Figures S2–S10

Percolation media	Pb(II) retention		Pb(II) vs Cd(II)/Co(II)		Pb(II) selectivity	
	OS1	OS2	OS1	OS2	OS1 vs OSc1	OS2 vs OSc1
PBS1: Na_2_HPO_4_ (10 mM), KH_2_PO_4_ (1.8 mM), NaCl (137 mM), KCl (2.7 mM), pH 6.6	+	+	+	+	−	−
PBS2: NaH_2_PO_4_ (25 mM) + Na_2_HPO_4_ (25 mM) + NaCl (150 mM), pH 6.6	−	−	−	−	−	−
HEPES (30 mM pH 6.5)	+	+	−	−	−	−
MES (50 mM pH 6.0)	+	+	−	−	−	−
Tris–HCl (20 mM), KCl (25 mM), NaCl (50 mM), MgCl_2_ (5 mM), pH 7.4	+	+	+	+	−	−
CH_3_COONa 10mM + NaClO_4_ 10 mM, pH 6.6	+	±	−	−	+	+
NaCl (10 mM pH 6.0)	+	+	−	−	−	−
NaNO_3_ (10 mM pH 6.0)	±	±	−	−	−	−
MgCl_2_ (5 mM pH 6.0)	+	+	+	+	+	+

Due to the number of tested percolation media and the need to measure ion content in all collected fractions, each extraction was performed only once. In a first step, only the C1 control was included to further reduce the overall duration of the procedure. This allowed a first assessment of the medium’s effect on ion retention. Regarding retention, a positive sign indicates Pb(II) retention on the specific support until elution. Regarding the behavior of Pb(II) vs Cd(II)/Co(II), a positive sign indicates retention of Pb(II) until elution and loss of Cd(II) and Co(II) before elution. Regarding Pb(II) selectivity, a positive sign indicates retention of Pb(II) only on the specific support, while a negative sign indicates retention on both specific and control supports. The retention on the second control sorbent will be examined once a given medium appears sufficiently promising to justify more extensive testing.

The use of phosphate-buffered saline (PBS) was commonly described in studies related to the development of aptasensors for lead ions [11, 13–15, 17, 18, 21, 27, 31, 32], although detailed composition was often unspecified. Therefore, two PBS solutions with different compositions were tested, as detailed in the Table 2 caption. During ion percolation in spiked PBS1, Pb(II) is retained, and specificity was observed (no retention of Co(II) and Cd(II) on OS1 and OS2) but no selectivity (retention of Pb(II) also on OSc1), whereas PBS2 does not allow Pb(II) retention on OS1 and OS2 (Figures S2–S3). By comparing the two PBS compositions, we can hypothesize that K^+^ promotes Pb(II) retention by favoring an adequate conformation of aptamers and then affinity for Pb(II).

HEPES and MES media, also implicated in the development of aptasensors [19, 23, 33], favor the retention of Pb(II) on OS1 and OS2, but neither specificity nor selectivity was observed (Figures S4–S5). The Tris buffer [10, 12, 16, 20, 22, 30, 34, 35] led to a retention profile similar to that obtained with PBS1, i.e., retention for Pb(II) without selectivity, likely due to KCl content (Fig. S6). However, it should be noted that it is important to use a control sorbent in parallel to accurately assess the selectivity of the oligosorbent as illustrated with results obtained using PBS or Tris–HCl solutions, which allow specificity for Pb(II) as Co(II) and Cd(II) were not retained but not selectivity, which could ultimately lead to the co-retention of other non-targeted ions by non-specific interactions. The sodium acetate/sodium perchlorate led to a different SPE profile, where Pb(II) was retained on OS1 and only slightly on OSc1, resulting in selective but not entirely specific retention (Fig. S7).

Single-salt solutions were also tested as percolating media to evaluate cation effects (Figures S8–S10) on ion retention. Only 5 mM MgCl_2_ provided both specificity and selectivity, emphasizing the crucial role of certain cations, particularly Mg^2+^, already known for stabilizing G quartet structures. However, using a MgCl_2_ solution as percolation and washing medium, Pb(II) recovery was only 60% when using 1 mL of 1 mM EDTA for elution. An increase in the elution volume from 1 to 5 mL allowed to achieve full recovery. An increase of the washing volume from 1 to 5 mL also allows the improvement of selectivity (Fig. S11). Under these last conditions of washing and elution, Pb(II) is extracted with yields of 77% and 75% on OS1 and OS2, respectively, compared to only 10% on the control sorbents OSc1 and OSc2, confirming the selective retention of Pb(II) on OS1 and OS2 mediated by G-quadruplex. However, 5 mL of 1 mM EDTA was required for elution, leading to dilution of the extract and thus an enrichment factor less than 1, indicating the need for further optimization of the elution conditions.

### Optimization of the elution conditions

**Fig. 1:**
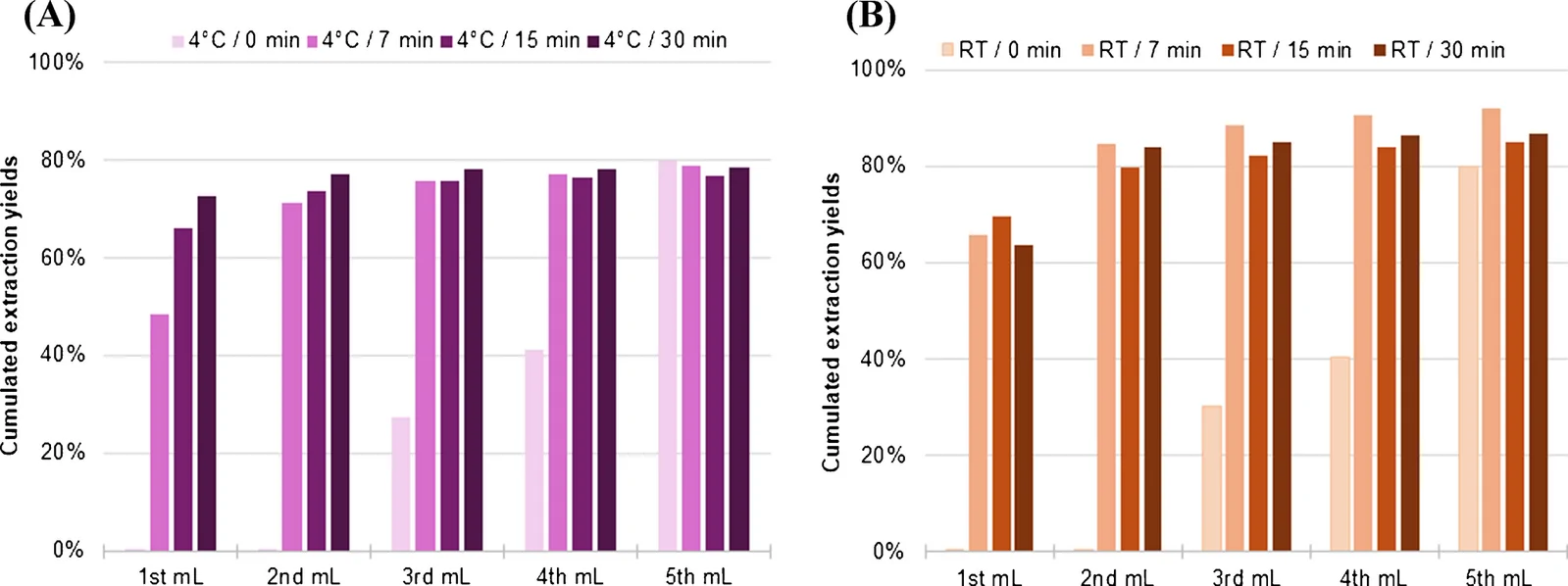
Cumulated extraction yields obtained in the elution fractions from OS1 after 0, 7, 15, or 30 min of stop flow during elution with fractions of 1 mM EDTA (pH 6.0) at 4 °C (**A**) or room temperature (RT) (**B**). Percolation: 1 mL of 5 mM MgCl_2_ spiked with 30 ng of Pb(II) (30 µg/L); washing: 5 mL of 5 mM MgCl_2_

In order to assess whether a kinetic limitation could explain this large volume of EDTA used to elute Pb(II) from the oligosorbents, immediate elution by passing 5 × 1 mL EDTA pH 6.0 through the OS1 by gravity was compared with elution using step flows, i.e., contact of the eluate for 7, 15, or 30 min for each 1 mL fraction at 4 °C. Figure 1 presents the extraction yields from cumulative elution fractions. A slow elution kinetic was observed. Indeed, without a stop flow, Pb(II) is only eluted from the third mL onwards, whereas stopping the flow for 7 min per fraction allowed elution from the first mL onwards and longer contact times increased recovery in the first fraction. All this demonstrates that there is a kinetic issue with this OS. It is worthwhile to notice that it was not the case with our previous study dealing with OSs dedicated to cadmium [28]. It has notably been observed in the literature that the G-quadruplex structure formed in the presence of Pb(II) is particularly stable, and notably more stable than with other ions that can stabilize the G-quadruplex form, such as Na^+^ or K^+^ [29].

Temperature also influenced kinetic. Indeed, as shown by the results reported in Fig. 1, increasing the temperature of the elution solution from 4 °C to room temperature (RT) accelerates elution of Pb(II) from OS1, allowing a compromise between extraction efficiency and duration of the procedure. The same behavior can be observed in OS2 in Fig. S12. Thus, at RT and with a stop flow of 7 min, a yield of more than 80% is obtained from the second mL.

**Fig. 2:**
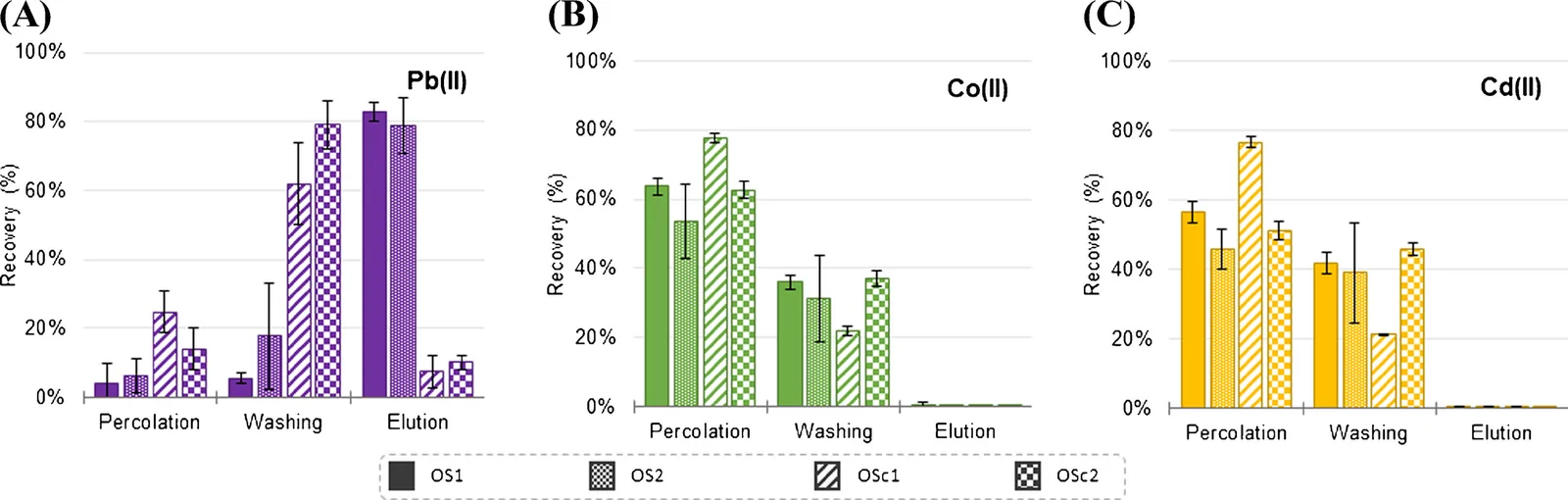
Extraction profiles on OS1, OS2, and the control sorbents, OSc1 and OSc2 of Pb(II) (**A**), Co(II) (**B**), and Cd(II) (**C**). SPE procedure: percolation: 1 mL of 5 mM MgCl_2_ spiked with 30 ng of each ion (30 µg/L); washing: 5 mL of 5 mM MgCl_2_; elution: 3 × 250 µL of 1 mM EDTA, pH 6.0, after each time a 7-min stop flow at room temperature

In order to improve the enrichment factor of the extraction procedure by reducing the elution volume, additional experiments combining stop flow and elution at RT were performed. Optimized conditions were established as 3 × 250 µL EDTA fractions, each brought into contact for 7 min at RT. These conditions were applied to the extraction of Pb(II) in the presence of the two other ions, Co(II) and Cd(II), and the results are reported in Fig. 2. Similar extraction profiles were obtained for Pb(II) using both specific OSs. These conditions yielded average recovery rates of Pb(II) (*n* = 3) of 83% ± 3% for OS1 and 79% ± 8% for OS2, compared to 7% ± 5% and 10% ± 2% for the control sorbents OSc1 and OSc2 in the combined elution fractions (750 µL). In return, as expected, Cd(II) and Co(II) were removed during the percolation and the washing step. Therefore, this SPE procedure promotes both selectivity and specificity for the extraction of Pb(II) on the OSs.

**Fig. 3:**
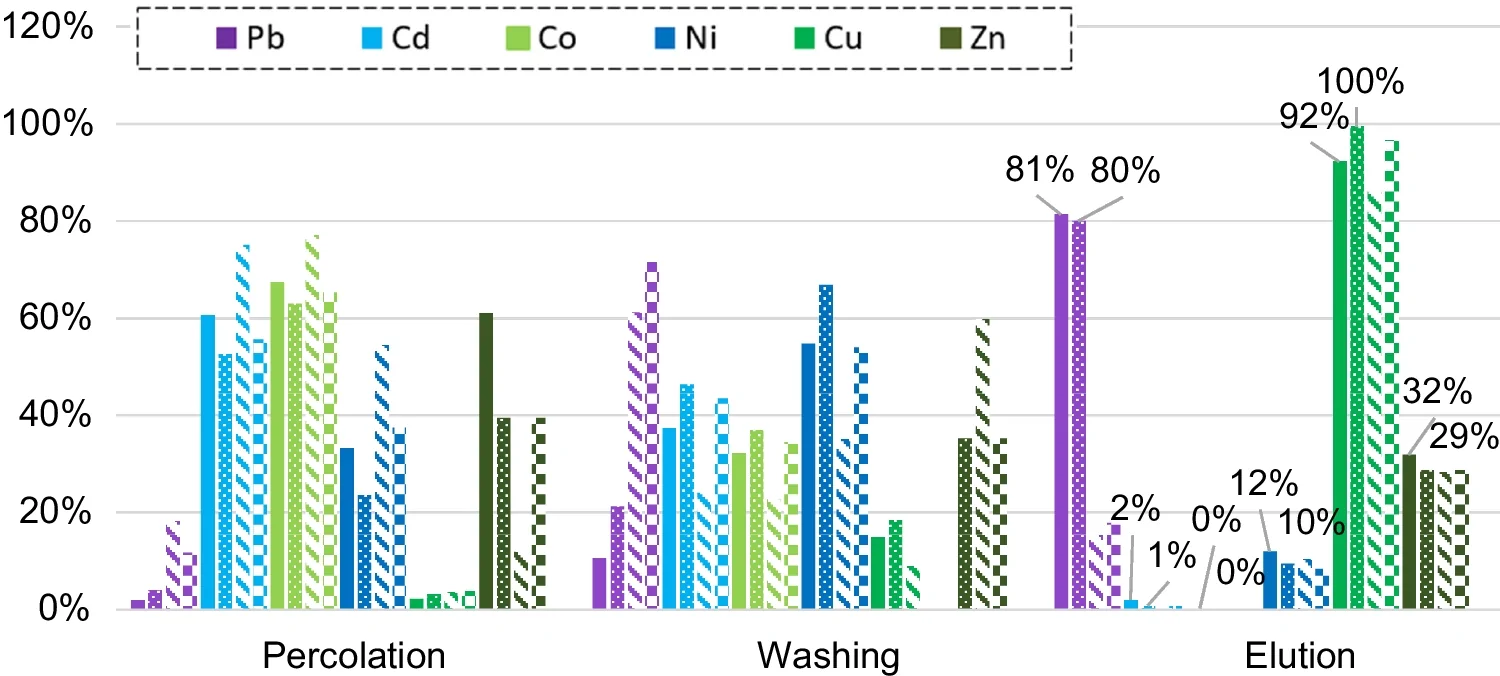
Extraction profiles of Pb(II) and other ions (Cd(II), Co(II), Ni(II), Cu(II), and Zn(II)) on OS1 (full histograms), OS2 (dotted histograms), OSc1 (hatched histograms), and OSc2 (square histograms). SPE procedure: percolation: 1 mL of MgCl_2_ 5 mM spiked with 30 ng of Pb(II), Cd(II), Co(II), Ni(II), Cu(II), and Zn(II); washing: 5 mL of the percolation solution; elution: 3 × 250 µL of EDTA 1 mM. Waiting time of 7 min at room temperature

Specificity was next studied with three additional ions, Ni(II), Zn(II), and Cu(II). Figure 3 presents the resulting extraction profiles. It appears that Ni(II) and Zn(II) are also quantitatively removed during the percolation and washing steps with the two specific OSs. Interestingly, Cu(II) shows a different behavior, being retained until the elution step. However, its retention is similar for all supports, indicating that this effect likely arises from non-specific interactions with the sorbent rather than specific interactions with the aptamers targeting Pb(II). These results further support the specificity of the proposed system while also highlighting potential limitations related to non-specific interactions for some elements for which another grafting sorbent may be considered. Moreover, our primary focus was on assessing the extraction of Pb(II) on our support. However, further investigations could explore the selective elution of Pb(II) while Cu(II) remains on the support, given the fundamentally different nature of the specific and non-specific interactions involved, thereby achieving Pb(II) specificity.

### Evaluation of the experimental capacity

**Fig. 4:**
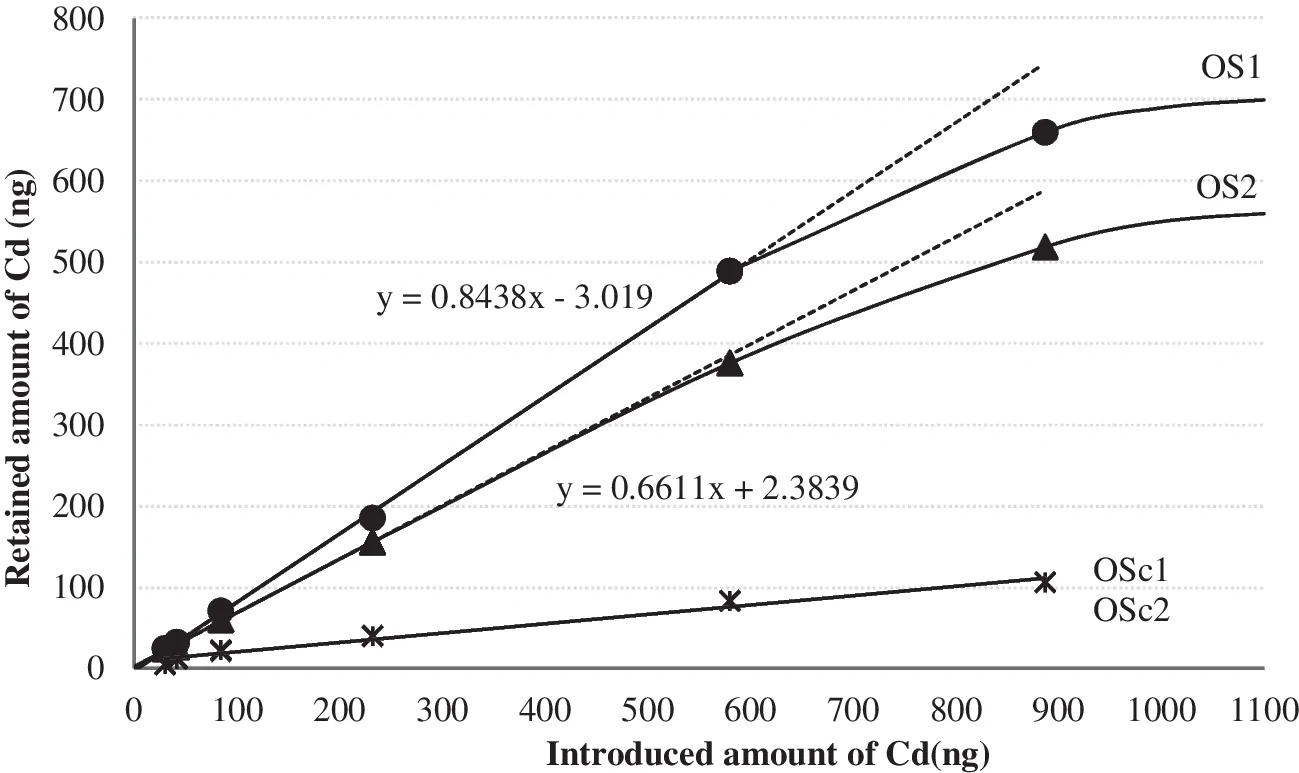
Capacity curves obtained after the percolation of an increasing amount of Pb(II) on OS1 (circle), OS2 (triangle), OSc1 (Cross), and OSc2 (diamond, superimposed with cross). The retained amount of Pb(II) corresponds to the amount found in the combined elution fractions after applying the optimized procedure (see Fig. 2 for extraction conditions)

Once the optimal extraction conditions were fixed, the capacity of the two specific OSs was investigated. It corresponds to the maximum amount of analyte that can be retained by specific interactions on a given amount of OS. To evaluate this parameter, the optimized SPE procedure was applied to solutions containing MgCl_2_ and spiked with increasing amounts (30–900 ng) of Pb(II). The amounts of Pb(II) retained on each OS and on the control sorbents, corresponding to the amount found in the combined elution fractions, are reported in Fig. 4. For OS1, the slope obtained up to 600 ng of Pb(II) percolated corresponds to a yield of 84%, which is similar to that measured after optimization of the elution conditions (83% ± 3%). When percolating 900 ng of Pb(II) on OS1, the extraction yield drops to 74%, indicating that the support’s capacity has been reached. Therefore, the experimental capacity of OS1 ranges between 600 and 900 ng. For OS2, a constant yield of 66% is observed until 250 ng percolated, which is slightly lower than the extraction yields obtained after the optimization (79 ± 8%). For 600 and 900 ng, the extraction yields drop to 64% and 58%, respectively. The capacity of OS2 support is thus between 250 and 600 ng. In conclusion, the capacity of OS1 is higher than that of OS2. For the OSc1 and OSc2 controls, the slopes of the superimposed curves correspond to recoveries of about 12% due to non-specific interactions. These results are in good agreement with extraction recoveries lower than 20% observed in Fig. 2, illustrating a low contribution of non-specific interactions in the retention of Pb(II) on the OSs. The OS1 support, characterized by its higher capacity, was then selected for the extraction of lead ions from real samples.

### Oligoextraction of Pb(II) from water samples

#### Oligoextraction of Pb(II) from tap water

**Fig. 5:**
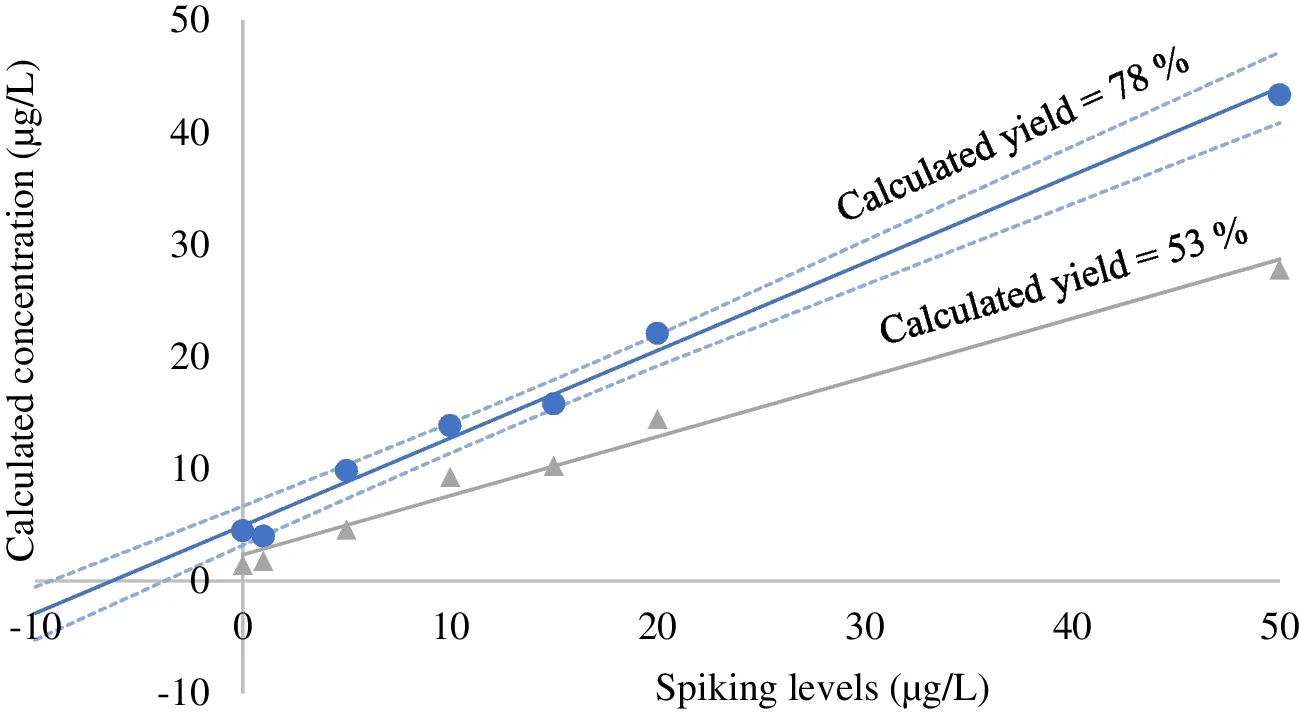
Calibration curve for the analysis of Pb(II) in tap water (solid line) and the 95% confidence hyperbolas (dotted lines) after oligoextraction in blue (round markers) or a simple dilution step (1/10) with 2% HNO₃ in gray (triangle markers); see Fig. 2 for extraction conditions

The concentration of Pb(II) in tap water from Paris was determined using the standard addition method. First, a small volume of a highly concentrated solution of MgCl_2_ spiked or not with Pb(II) was added to the tap water to reach final concentrations of 5 mM for MgCl_2_ and between 0 and 50 µg/L for Pb(II). This allows MgCl_2,_ which is necessary for retaining Pb(II) on the OS, to be introduced while limiting the dilution of tap water to a factor of only 1.01. Extractions on OS1 were next carried out for the different concentration levels of Pb(II) in tap water, and for each SPE, the combined elution fractions were analyzed by ICP-MS. The resulting calibration curve is reported in Fig. 5.

To determine the initial concentration of lead in the tap water, the calibration line was extrapolated to its intersection with *y* = 0. In addition, confidence hyperbolas were plotted to establish a 95% confidence interval for the calculated concentration. Thus, the lead concentration obtained is 6.3 µg/L, with a 95% confidence interval ranging from 3.8 to 9.3 µg/L. Since the regulatory limit is set at 10 µg/L, the values obtained are consistent with the expected range. Moreover, based on the slope of the calibration curve, the extraction efficiency was determined to be 78%, close to that obtained in spiked ultra-pure water containing MgCl_2_ 5 mM (83% ± 3%, *n* = 3). The similarity of both values indicates that the extraction recovery of lead on the OS is not affected by matrix effects. Tap water samples spiked with Pb(II) were also simply diluted (1/10) in 2% HNO₃ to be directly analyzed by ICP-MS without passing through the OS. The calibration curve is characterized by a slope of 53%, thus indicating a matrix effect in ICP-MS analysis for tap water using this simple dilution step. The resulting concentration was calculated to be 2.1 µg/L, which is lower than the value obtained using the oligoextraction procedure. This demonstrates the benefit of the oligoextraction step, which prevents underestimation of the initial Pb(II) concentration present in the sample. Increasing the dilution ratio would reduce the risk of matrix effects, but sensitivity would be reduced and trace analysis compromised.

#### Oligoextraction of Pb(II) from Seine River water

**Fig. 6:**
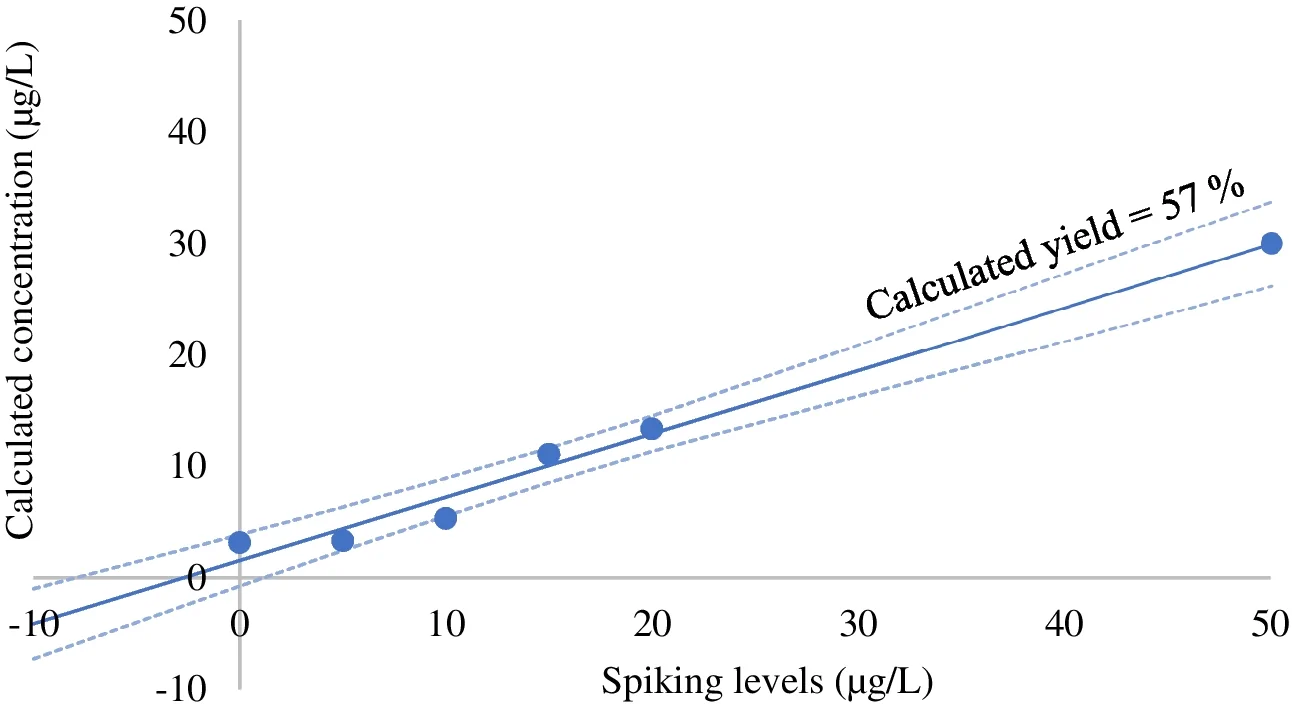
Calibration curve for the analysis of Pb(II) in river Seine water (solid lines) and the 95% confidence hyperbolas (dotted lines) after oligoextraction (see Fig. 2 for extraction conditions)

To go further, a more complex matrix was studied, i.e., the river Seine water. Prior to the oligoextraction step, a filtration was performed. Subsequently, a small volume of a highly concentrated solution of MgCl_2_ spiked or not with Pb(II) was added to the sample to reach final concentrations of 5 mM for MgCl_2_ and between 0 and 50 µg/L for Pb(II). Extractions on OS1 were next carried out, followed by ICP-MS analysis. The resulting calibration curve is reported in Fig. 6. Thanks to the standard addition method, an initial Pb(II) concentration of 2.7 µg/L was determined, ranging from 1.2 to 7.9 µg/L with a 95% confidence interval, that is consistent with the regulatory limit set at 14 µg/L.

Moreover, based on the slope of the calibration curve, the extraction recovery was determined to be 57%, which is lower than that obtained in spiked ultra-pure or tap waters containing MgCl_2_ 5 mM. A reaction between lead and some substances present in the Seine water, leading to the formation of a complex, could explain the reduced affinity between Pb(II) and the aptamers, thereby decreasing the retention strength and consequently the extraction yield. Despite this low yield, this oligoextraction step offers major advantages compared to the direct analysis of Seine River water. Indeed, with simple dilution (1/10) of the Seine River water with 2% HNO₃, the quantity obtained is below the LOQ, making the quantification of lead at trace level less reliable in direct analysis with ICP-MS. Reducing the dilution ratio to 1/5 would allow the sample to be quantified. However, such a low dilution for a complex sample would result in a strong matrix effect, such as ionization suppression and the formation of spectral interferences, which deteriorate the sensitivity and accuracy of the measurements for diluted river Seine samples. Moreover, the presence of these compounds increases the risk of cone and torch fouling, leading to reduced signal stability and more frequent instrument maintenance.

### Oligoextraction of Pb(II) from serum samples

**Fig. 7:**
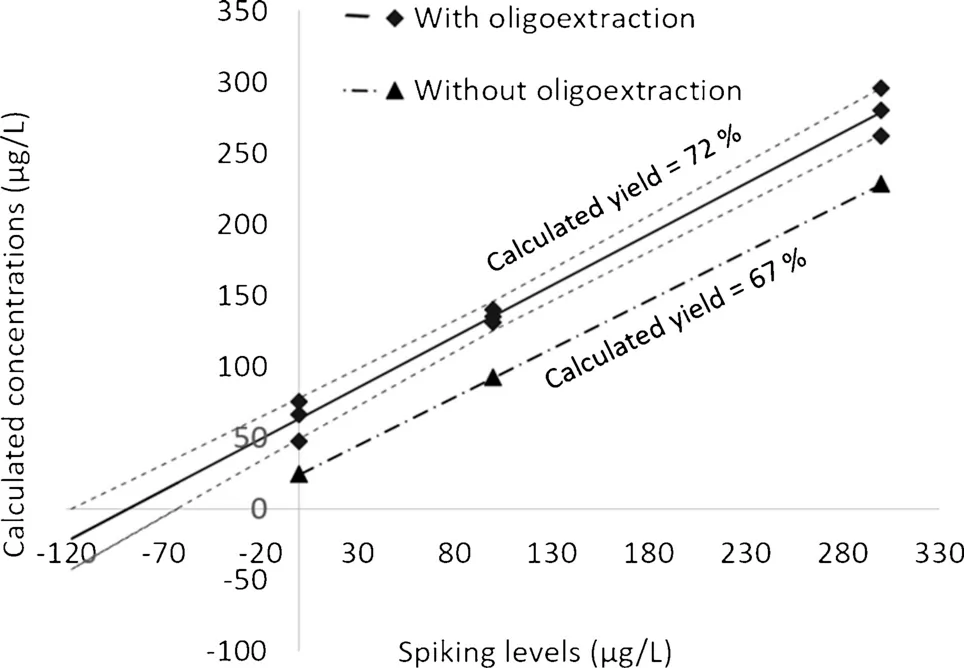
Calibration curves for the analysis of Pb(II) in human serum submitted to an acid digestion step and with (solid lines and square markers) or without (dotted lines and triangle markers) oligoextraction on OS1. Spiking levels in serum are between 0 and 300 µg/L. The 95% confidence hyperbolas for the results with oligoextraction are shown as dotted lines

Prior to oligoextraction, an acid digestion procedure was achieved to eliminate proteins that might form complexes with Pb(II) and obstruct the SPE cartridge [36]. The digested serum was further centrifuged, and the resulting supernatant was either mixed with a solution of MgCl_2_ to be percolated on OS1 for achieving a sample clean-up step or simply diluted (dilution rate of 60 to obtain the same dilution factor as after the oligoextraction) with a 2% HNO_3_ solution before being analyzed by ICP-MS. This protocol was applied to non-spiked and spiked human serum at concentrations ranging from 100 to 300 µg/L (*n* = 3 for each concentration level). The results obtained using OS1 are provided in Fig. 7. The initial Pb(II) concentration in serum was determined in the same manner as previously described for tap water. Hyperbolas representing a 95% confidence interval were included to quantify the uncertainty on both sides of the untreated serum value. The Pb(II) concentration in non-spiked serum was found to be 87 µg/L, with a 95% confidence interval ranging from 62 to 117 µg/L. The slope of the curve facilitated the assessment of the extraction yield, resulting in a value of 72% with a standard deviation of 3.2% (*n* = 7). This extraction yield is slightly lower than that obtained previously for spiked ultra-pure water containing MgCl_2_ 5 mM. This very small difference could be attributed to low matrix effect during the extraction procedure on OS that slightly affects the recoveries, but it could also be attributed to some loss of Pb(II) during the acid digestion step, as well as the accompanying centrifugation steps. To confirm this hypothesis, doping was performed after the acid digestion stage, which increased the yield to 74%. For the analysis of Pb(II) in the diluted supernatant by ICP-MS without a preliminary oligoextraction step, a recovery rate of 67 ± 0.2% was calculated, thus indicating again a matrix effect on ICP-MS analysis despite the dilution factor of 60. Furthermore, the initial concentration in the serum was determined to be 35 µg/L (confidence interval ranging from 34 to 37 µg/L) without oligoextraction compared to 87 µg/L with oligoextraction (confidence interval ranging from 62 to 117 µg/L). Moreover, by applying the dilution factors and yields obtained to the instrumental LOQ, a value of 2.8 µg/L is calculated for the determination of Pb(II) in serum when using oligoextraction compared to 4.0 µg/L without oligoextraction. Thus, the oligoextraction step helps to reach better LOQ and to prevent from fouling the ICP-MS.

Table 3 summarizes the extraction recovery values of Pb(II) determined for the different tested samples without or with oligoextraction. These results demonstrate that oligoextraction significantly improves the recovery of lead across different matrices, highlighting the effectiveness of the developed method. In this study, the method accuracy was assessed through spiking experiments and comparison with direct ICP-MS analysis. The use of CRMs will be considered in future work to further strengthen validation.

**Table 3:** Recovery of Pb(II) from different matrices with and without oligoextraction. Notes: The symbol “-” indicates that the recovery without oligoextraction was not measured for river water

Matrix	Recovery without oligoextraction (%)	Recovery with oligoextraction (%)
Tap water	53	78 ± 3 (*n* = 7)
River water	-	57 ± 4 (*n* = 6)
Serum	67	72 ± 3 (*n* = 3)

### Repeatability, stability, and storage

The aptamer grafting procedure was repeated multiple times under identical conditions, and the functionalized supports obtained consistently exhibited similar immobilization efficiencies and analytical responses, confirming the robustness and reproducibility of the grafting step. Experiments to optimize the extraction medium (more than 60 extractions), as well as more than fifteen serum extractions (more than 15 extractions), were performed using a single cartridge that remained in service for 18 months without any detectable decline in extraction performance. Similar results, obtained for a second cartridge used for more than 15 extractions in real environmental matrices, highlight the high stability of the OSs submitted to real samples. All sorbent cartridges were stored in water at 4 °C when not in use to ensure long-term stability.

### Comparison and discussion with other studies

As summarized in Table 4, a wide variety of sorbents have already been reported for the extraction and preconcentration of Pb(II), including commercial chelating resins such as Analig ME‑02, polymeric supports like XAD‑4, task-specific ionic liquid‑modified carbon nanotubes (TSIL‑CNTs), metal–organic frameworks (MOF Ag12), and ion-imprinted polymer (IIP). All SPE procedures with these sorbents provide high recoveries (typically ≥ 90%) for Pb(II) present in different kinds of water or other complex matrices. However, for the studies implementing commercial sorbents, TSIL-CNTs, and MOF, specificity was assessed by monitoring the effect of common inorganic ions on Pb(II) recovery, without systematically measuring the retention of these competing ions on the sorbent. None of them has studied selectivity, even with sorbents presented as more selective such as MOF, so that the actual contribution of non-specific interactions remains largely unknown. In contrast, selectivity was successfully demonstrated in the case of the IIP sorbents, comparing the retention of Pb(II) on the IIP and its corresponding non-imprinted polymer (NIP). It is worth noting that, for IIPs, specificity was evaluated by examining the effect on the extraction recovery of Pb(II) in the presence or absence of interfering ions, rather than their retention on the supports. Regarding reusability and repeatability, our OS was monitored for over 18 months, with at least 60 extractions carried out on the same support, and very good stability in lead extraction recovery was observed. This has also been the case in some other studies, but over much shorter periods or with a much smaller number of uses. To the best of our knowledge, no previous study has reported a solid-phase extraction procedure on OS cartridge using immobilized aptamers for the selective extraction of Pb(II), making the present work the first demonstration of such an approach.

**Table 4:** Comparison of reported solid-phase extraction sorbents for Pb(II)

Support	Specificity/selectivity	Matrix	Recovery (%)	Reusability (for recovery)	Ref.
Analig ME-02	NA/NA	Pure media	≥ 93%	NA	[37]
XAD-4	Na^+^, K^+^, Mg^2+^, Ca^2+^, SO_4_^2−^/NA	Water, urine	≥ 92%	Inter- and intra-day, *n* = 6 (NA)	[38]
TSIL-CNTs	K^+^, Ca^2+^, SO_4_^2−^, Cl^−^, CO_3_^2−^, NO3^−^, Mg^2+^, Ba^2+^, Al^3+^, Ni^2+^, Au^3+^, Ag^+^, Tl^+^, Cd^2+^, Zn^2+^, Fe^2+^, Cu^2+^/NA	CRM (CCU-1b), red lipstick, pine leaves, water	97.3–98.6%	NA	[39]
MOF Ag12	Alkaline, alkaline earth, transition metals/NA	Distilled, tap, river, lake, waste, and seawater	99%	*n* = 5	[40]
IIP	Ca^2+^, Mg^2+^, Cd^2+^, Cr^3+^, Cu^2+^/NIP	Infant formula	90.0%	Inter- and intra-day, *n* = 3 (RSD ≤ 5% for both)	[41]
IIHC	Cd^2+^, Cu^2+^, Zn^2+^, Co^2+^, Mn^2+^, Cr^3+^, Fe^3+^/NIC	CRM (DORM-3, MESS-3, PACS-2), water, parenteral solutions, urine	92.7–110.0%	*n* = 10 (RSD ≤ 5%)	[42]
Aptamers	Cd^2+^, Co^2+^, Ni^2+^, Cu^2+^, Zn^2+^/others aptamers	River and tap water, serum	83%	18 months, *n* ≥ 60 (RSD 3%)	This work

*NA* non available

## Conclusion

The aim of this study was to develop an aptamer-functionalized extraction sorbent for the selective and specific extraction of Pb(II). The two sequences identified in the literature as specific for Pb(II) were grafted on a solid phase to obtain oligosorbents that have demonstrated their potential for the selective extraction of this cation. Control sorbents composed of non-specific aptamers were prepared and used to evaluate the actual selectivity of the oligoextraction process by verifying that these supports did not retain Pb(II). The specificity of the oligosorbents was evaluated by verifying that other cations, Cd(II) and Co(II), were not retained on the OSs. This study also showed the strong effect of the nature of the percolation medium, particularly the effect of cations, such as Mg^2+^, on selectivity and specificity. After optimizing the extraction procedure in pure media and having determined the capacity, oligoextraction was applied to the determination of Pb(II) in real samples. These experiments showed that extraction recoveries obtained with tap water and serum samples were close to those obtained in pure media, thus indicating that extraction recoveries on the OSs were not affected by matrix components. Furthermore, comparison of the results obtained by ICP-MS for the same samples that underwent a simple dilution step, replacing the oligoextraction step, showed significant differences in the concentrations measured for different types of samples (drinking water, river water, human serum digests). Several prospects can be envisaged for these media in the future, such as their application to other types of highly complex matrices, such as food matrices, in order to make analyses more reliable by eliminating matrix effects. Finally, given the high specificity of these OSs, it is possible to consider their use upstream of less specific but also less expensive instruments than ICP-MS, such as X-ray fluorescence or electrochemical sensors, to compensate for the lower specificity of these detection modes.

## Supplementary Information

Below is the link to the electronic supplementary material.

Supplementary file1 (DOCX 564 KB)

## Data Availability

All data are included in the paper.
