# Supplementary material for: Development of an aptamer-functionalized sorbent for the selective and specific extraction of lead from environmental and biological samples
Source: Anal Bioanal Chem. 2026 May 20;418(18):6101–13. doi: 10.1007/s00216-026-06561-8 (PMC13541864; doi:10.1007/s00216-026-06561-8)
Supplement: Supplementary file 1 [file 216_2026_6561_MOESM1_ESM.docx]

**Development of an aptamer-functionalized sorbent for the selective and specific extraction of lead from environmental and biological samples**

**Alice Taxil—Paloc^a,b^, Fanny Gignac ^a^, Nathalie Delaunay ^a^, Valérie Pichon ^a,b,*^**

^a^ Department of Analytical, Bioanalytical Sciences, and Miniaturization, UMR 8231 Chemistry, Biology and Innovation, ESPCI Paris, PSL University, CNRS, 10 rue Vauquelin, Paris 75005, France

^b^ Sorbonne Université, Paris 75005, France

Supplementary material

$$Limit of quantification= \frac{10 \sigma}{k}$$

**Equation S1.** equation for calculating the limit of quantification. σ is the standard deviation in counts per second (cps) of 10 blank solutions (in 2% HNO_3_) and k the sensitivity of the instrument (cps/(µg/L))

**Table S1.** LOQ of ^59^Co, ^208^Pb, ^63^Cu, ^60^Ni and ^66^Zn in 2% HNO_3_ determined by ICP-MS using Equation S1

| **Element** | **LOQ (ng/L)** |
| --- | --- |
| Cobalt | 1 |
| Lead | 45 |
| Copper | 51 |
| Nickel | 44 |
| Zinc | 224 |

| Theorical concentration (µg/L) | Measured concentration (µg/L) |
| --- | --- |
| 50 | 48 |
| 100 | 99 |
| 500 | 506 |
| 1000 | 940 |
| 3000 | 2873 |
| 5000 | 4802 |
| 10000 | 9630 |

**Figure S1.** typical calibration curve for ^208^Pb obtained by analyzing in ICP-MS solutions of Pb(II) in HNO_3_ 2% with concentrations between 50 and 10000 µg/L

**Figure S2.** Extraction profiles obtained when percolating PBS1 (Na_2_HPO_4_ (10 mM) + KH_2_PO_4_ (1.8 mM) + NaCl (137 mM) + KCl (2.7 mM), pH 6.6) spiked with Pb(II), Cd(II) and Co(II) on OS1, OS2 and OSc1. SPE procedure: Percolation: 1 mL of Na_2_HPO_4_ (10 mM) + KH_2_PO_4_ (1.8 mM) + NaCl (137 mM) + KCl (2.7 mM)) spiked with 30 ng of Pb(II), Cd(II) and Co(II); Washing: 4 mL of the percolation buffer; Elution: 2 mL of EDTA 1 mM + NaCl 50 mM + Tris HCl 10 mM, pH 7.0

**Figure S3.** Extraction profiles obtained when percolating PBS2 (Na_2_HPO_4_ (25 mM) + NaH_2_PO_4_ (25 mM) + NaCl (150 mM), pH 6.6) spiked with Pb(II), Cd(II) and Co(II) on OS1, OS2 and OSc1. SPE procedure: Percolation: 1 mL of Na_2_HPO_4_ (25 mM) + NaH_2_PO_4_ (25 mM) + NaCl (150 mM) spiked with 30 ng of Pb(II), Cd(II) and Co(II); Washing: 1 mL of the percolation buffer; Elution: 2 mL of EDTA 1 mM + NaCl 50 mM + Tris HCl 10 mM, pH 7.0

**Figure S4.** Extraction profiles obtained when percolating HEPES (30 mM, pH 6.5) spiked with Pb(II), Cd(II) and Co(II) on OS1, OS2 and OSc1. SPE procedure: Percolation: 1 mL of HEPES (30 mM) spiked with 30 ng of Pb(II), Cd(II) and Co(II); Washing: 2 mL of NaNO_3_ (10 mM); Elution: 2 mL of EDTA 1 mM + NaCl 50 mM + Tris HCl 10 mM, pH 7.0

**Figure S5.** Extraction profiles obtained when percolating MES (50 mM, pH 6.0) spiked with Pb(II), Cd(II) and Co(II) on OS1*,* OS2 and OSc1. SPE procedure: Percolation: 1 mL of MES (50 mM) spiked with 30 ng of Pb(II), Cd(II) and Co(II); Washing: 2 mL of the percolation buffer; Elution: 2 mL of EDTA 1 mM + NaCl 50 mM + Tris HCl 10 mM, pH 7.0

**Figure S6.** Extraction profiles obtained when percolating Tris-HCl (20 mM) + KCl (25 mM) + NaCl (50 mM) + MgCl_2_ (5 mM), pH 7.4 spiked with Pb(II), Cd(II) and Co(II) on OS1*,* OS2 and OSc1. SPE procedure: Percolation: 1 mL of Tris-HCl (20 mM) + KCl (25 mM) + NaCl (50 mM) + MgCl_2_(5 mM) spiked with 30 ng of Pb(II), Cd(II) and Co(II); Washing: 1 mL of the percolation buffer; Elution: 3 mL of EDTA 1 mM + NaCl 50 mM + Tris HCl 10 mM, pH 7.0

**Figure S7.** Extraction profiles obtained when percolating sodium acetate (10 mM) + sodium perchlorate (10 mM), pH 6.0 spiked with Pb(II), Cd(II) and Co(II) on OS1, OS2 and OSc1. SPE procedure: Percolation: 1 mL CH_3_COONa 10mM + NaClO_4_ 10 mM, pH 6.6 spiked with 30 ng of Pb(II), Cd(II) and Co(II); Washing: 1 mL of the percolation solution; Elution: 2 mL of EDTA 1 mM + NaCl 50 mM + Tris HCl 10 mM, pH 7.0

**Figure S8.** Extraction profiles obtained when percolating NaCl (10 mM, pH 6.0) spiked with Pb(II), Cd(II) and Co(II) on OS1, OS2 and OSc1. SPE procedure: Percolation: 1 mL of NaCl (10 mM) spiked with 30 ng of Pb(II), Cd(II) and Co(II); Washing: 1 mL of the percolation solution; Elution: 3 mL of EDTA 1 mM + NaCl 50 mM + Tris HCl 10 mM, pH 7.0

**Figure S9.** Extraction profiles obtained when percolating NaNO_3_ (10 mM, pH 6.0) spiked with Pb(II), Cd(II) and Co(II) on OS1, OS2 and OSc1. SPE procedure: Percolation: 1 mL of NaNO_3_ (10 mM) spiked with 30 ng of Pb(II), Cd(II) and Co(II); Washing: 1 mL of the percolation solution; Elution: 1 mL of EDTA 1 mM + NaCl 50 mM + Tris HCl 10 mM, pH 7.0

**Figure S10.** Extraction profiles obtained when percolating MgCl_2_ (5 mM, pH 6.0) spiked with Pb(II), Cd(II) and Co(II) on OS1, OS2 and on their control sorbents. SPE procedure: Percolation: 1 mL of MgCl_2_ (5 mM) spiked with 30 ng of Pb(II), Cd(II) and Co(II); Washing: 1 mL of the percolation solution; Elution: 1 mL of EDTA 1 mM, pH 6.0

**Figure S11.** Extraction profiles obtained when percolating MgCl_2_ (5 mM, pH 6.0) spiked with Pb(II), Cd(II) and Co(II) on OS1, OS2 and on the control sorbents OSc1 and OSc2. SPE procedure: Percolation: 1 mL of MgCl_2_ (5 mM) spiked with 30 ng of Pb(II), Cd(II) and Co(II); Washing: 5 mL of the percolation solution; Elution: 5 mL of EDTA 1 mM, pH 6.0

Cumulated extraction yields

|  | 4°C / 0 min | 4°C / 7 min | 4°C / 15 min | 4°C / 30 min | RT / 0 min | RT / 7 min | RT / 15 min | RT / 30 min |
| --- | --- | --- | --- | --- | --- | --- | --- | --- |
| 1st mL | 0.228137% | 53.8937% | 72.2692% | 74.4833% | 0.228137% | 79.491% | 78.5197% | 74.4833% |
| 2nd mL | 0.760456% | 66.6303% | 75.37% | 77.1561% | 0.760456% | 88.735% | 81.5789% | 77.1561% |
| 3rd mL | 18.7072% | 72.3595% | 76.1452% | 77.6194% | 25.7605% | 90.6811% | 83.4211% | 77.6194% |
| 4th mL | 33.9163% | 73.6332% | 76.6032% | 78.1183% | 38.7605% | 95.7335% | 84.4408% | 78.1183% |
| 5th mL | 82% | 74.6157% | 76.8147% | 78.6885% | 80% | 97.006% | 85.2303% | 78.6885% |

**Figure S12.** Cumulated extraction yields obtained in elution fractions on OS2 with a waiting time of 0, 7, 15 or 30 min for each elution fraction and an elution solution (EDTA 1 mM, pH 6.0) at 4°C (blue histograms) or at room temperature (green histograms)
